# Phase-resolved functional lung MRI detects single-dose and sustained bronchodilator responses in COPD in a randomized crossover trial

**DOI:** 10.1007/s00330-026-12604-6

**Published:** 2026-05-20

**Authors:** Andreas Voskrebenzev, Till F. Kaireit, Marius M. Klein, Agilo L. Kern, Lea Behrendt, Filip Klimeš, Robin A. Müller, Thomas Kayser, Frank Wacker, Jens Vogel-Claussen, Jens M. Hohlfeld

**Affiliations:** 1 Institute of Diagnostic and Interventional Radiology, Hannover Medical School, Hannover, Germany; 2 Biomedical Research in Endstage and Obstructive Lung Disease Hannover (BREATH), German Center for Lung Research (DZL), Hannover, Germany; 3 Department of Diagnostic and Interventional Radiology, Charité Universitätsmedizin Berlin, Corporate Member of Freie Universität Berlin and Humboldt-Universität zu Berlin, Berlin, Germany; 4 Fraunhofer Institute of Toxicology and Experimental Medicine, Hannover, Germany; 5 Department of Respiratory Medicine and Infectious Disease, Hannover Medical School, Hannover, Germany

**Keywords:** Bronchodilation, COPD, Functional pulmonary proton MRI, Lung, PREFUL

## Abstract

**Objectives:**

To evaluate the effects of tiotropium/olodaterol (T/O) on phase-resolved functional lung (PREFUL) MRI parameters in hyperinflated chronic obstructive pulmonary disease (COPD) patients and examine correlations with conventional cardiopulmonary and hyperpolarized ^129^Xe MRI measures.

**Materials and methods:**

Retrospective subanalysis of a prospective, randomized, placebo-controlled, crossover trial with open-label extension. Thirty-two patients with moderate-to-severe COPD (61.5 ± 7.7 years; 17 men); 30 completed the MRI extension at 1.5 T. PREFUL analysis yielded regional ventilation (RVent), flow-volume loop correlation metric (FVL-CM), normalized perfusion (QN), ventilation defect percentage (VDP), perfusion defect percentage (QDP), V/Q match metrics (VQM), and pulmonary pulse wave velocity (PWV; post-hoc parameter). Linear mixed-effects models tested treatment effects; correlations were evaluated with Spearman’s rank and bootstrap 95% confidence intervals (95% CIs).

**Results:**

PREFUL parameters improved after T/O single dose (SD) versus placebo, including improvements in FVL-CM by 4.1 percentage points (pp; 95% CI: 1.0 to 7.3 pp) and QN by 0.4 pp (95% CI: 0.2 to 0.6 pp) and reductions in VDP and QDP, with parallel gains in VQM(Non-Defect) (*p* < 0.05). PWV decreased after multiple doses (−0.87 m/s, 95% CI: −1.26 to −0.48 m/s). PREFUL MRI baseline values showed significant correlations with pulmonary function tests, cardiac, dynamic contrast-enhanced and ^129^Xe MRI. SD treatment-induced absolute changes in VDP(FVL-CM) correlated with reductions in residual volume (*ρ* = 0.41, 95% CI: 0.02 to 0.64). Further correlations were observed between PREFUL MRI and ¹²⁹Xe-derived VDP, apparent diffusion coefficient, and compartment ratios.

**Conclusion:**

PREFUL MRI sensitively captured immediate SD T/O-induced improvements in V/Q parameters and dose-dependent PWV responses after sustained bronchodilation.

**Key Points:**

***Question*** *Can phase-resolved functional lung (PREFUL) MRI sensitively capture immediate single-dose and sustain multi-dose effects of tiotropium/olodaterol on ventilation-perfusion and vascular function in COPD patients?*

***Findings*** *Tiotropium/olodaterol improved PREFUL MRI-derived ventilation, perfusion, and V/Q matching parameters after a single dose, with sustained pulmonary vascular improvements after repeated dosing.*

***Clinical relevance*** *PREFUL MRI detected immediate and sustained functional improvements after tiotropium/olodaterol and showed significant correlations with cardiopulmonary tests and hyperpolarized ¹²⁹Xe MRI, supporting its role as a sensitive, radiation-free tool for monitoring COPD treatment response.*

**Graphical Abstract:**

**Figure:**
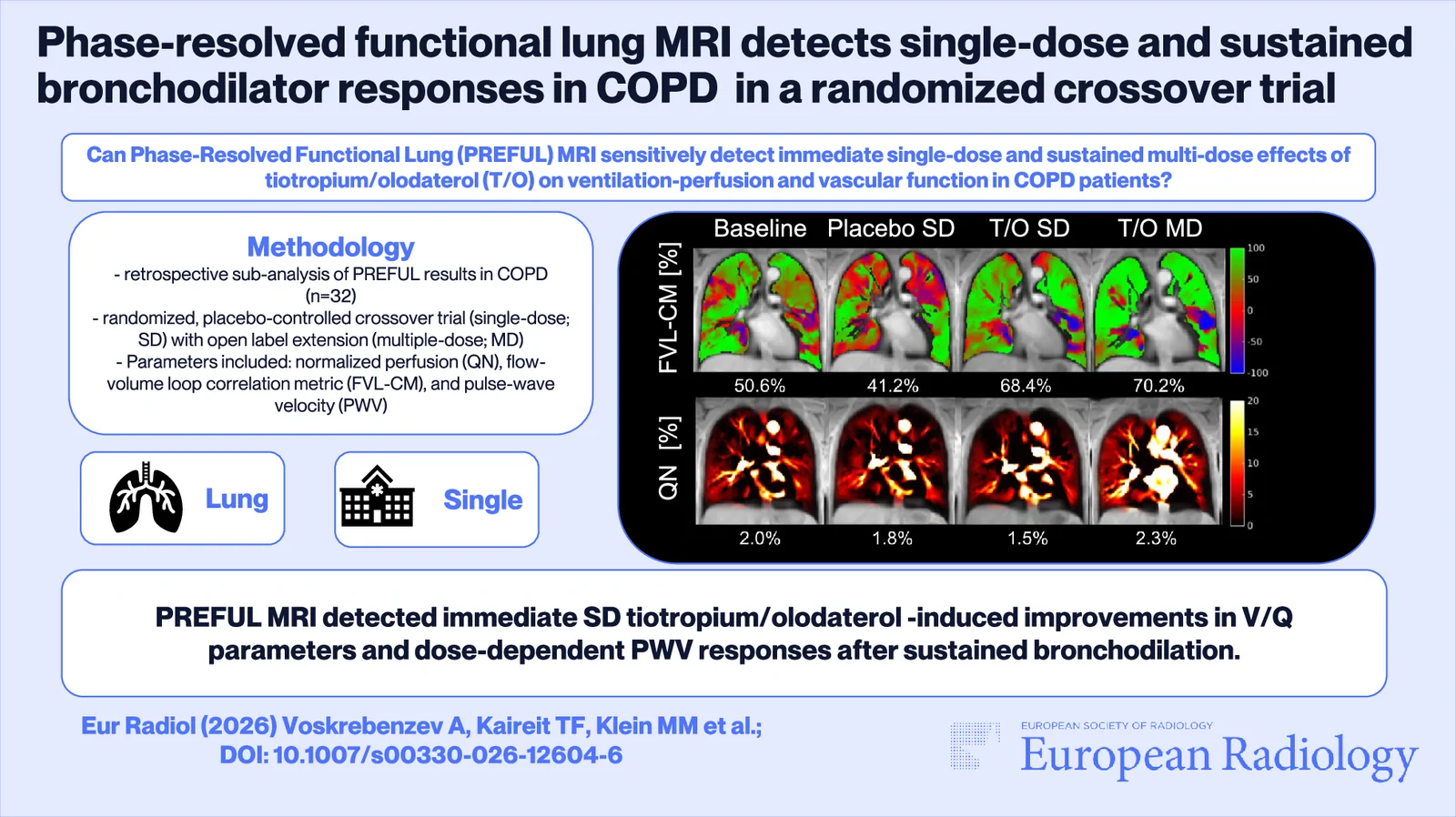

## Introduction

Chronic obstructive pulmonary disease (COPD) is a leading cause of morbidity and mortality worldwide [1] and is characterized by persistent airflow limitation, gas trapping, and progressive hyperinflation [2]. Hyperinflation increases intrathoracic pressure, imposes additional mechanical load on the respiratory muscles and exerts hemodynamic consequences by limiting venous return and impairing cardiac filling [3, 4]. Long-acting muscarinic antagonists (LAMA) and long-acting β₂-agonists (LABA) are established therapeutic strategies [5–7], and dual bronchodilation with tiotropium/olodaterol (T/O) has demonstrated improvements in lung function, symptoms, and exercise capacity in COPD patients [8, 9]. Beyond pulmonary mechanics, pharmacological lung deflation has been shown to improve biventricular filling and stroke volume, thereby establishing a link between airway obstruction and cardiovascular function [10, 11].

Magnetic resonance imaging (MRI) is increasingly used to characterize cardiopulmonary interactions in COPD [12]. Cardiac cine MRI permits accurate quantification of chamber volumes, mass, and EF, and has been used to demonstrate bronchodilator-induced increases in left ventricular end-diastolic volume index (LV-EDVi) and stroke volume index (SVi) [13]. On the pulmonary side, MRI techniques have historically relied on hyperpolarized ^3^He or ^129^Xe gas for ventilation mapping [14–20], and on contrast-enhanced methods for perfusion imaging [21–24]. Although highly informative, these approaches are constrained by cost, necessity for additional hardware, limited availability, and, in the case of gadolinium-based perfusion, raise concerns regarding repeated use [25].

Recent advances have enabled proton MRI methods that assess regional lung function during free breathing without exogenous contrast agents. Fourier decomposition and related techniques exploit the cyclic signal fluctuations during respiration and cardiac pulsation to generate surrogates of ventilation and perfusion [26–32]. These approaches were validated against more established techniques and thus offer a radiation-free and repeatable alternative for ventilation-perfusion (V/Q) matched functional imaging in COPD [33–39]. Moreover, dynamic ventilation assessment using a regional flow-volume loop correlation metric (FVL-CM) derived from phase-resolved functional lung (PREFUL) MRI has shown increased sensitivity in comparison with conventional ventilation measures, both in patients after lung transplantation [40, 41] and in COPD following bronchodilation [42]. PREFUL MRI therefore offers regional information on airway mechanics that is not captured by standard spirometry.

While free-breathing proton MRI has been validated against established lung function tests and gas-based MRI [33, 35, 43–45], evidence regarding its application in interventional COPD studies remains limited [42]. Previous interventional work has largely focused on spirometry, body plethysmography, and diffusion capacity as outcome measures, complemented by cardiac MRI in selected studies [6, 8–10]. Functional proton MRI may offer additional insight into how dual bronchodilation modifies regional lung function and VQ matching, thereby contributing to understanding cardiopulmonary interactions. Demonstrating such associations could further establish free-breathing proton MRI as a valuable biomarker for therapeutic studies.

The main results of the present randomized, placebo-controlled, investigator-blinded crossover trial with open-label extension have recently been reported [46]. In that analysis, single-dose (SD) T/O significantly reduced residual volume and improved spirometric outcomes, while sustained multiple-dose (MD) therapy was required to achieve significant increases in LV-EDVi. These findings highlight that bronchodilation exerts rapid pulmonary effects, whereas cardiovascular improvements require continued therapy.

The subanalysis presented here builds directly on those findings by investigating PREFUL MRI data in the same cohort, including regional ventilation (RVent) heterogeneity, FVL-CM, normalized perfusion (QN), and V/Q. We hypothesized that dual bronchodilation with T/O would improve PREFUL-derived regional ventilation, perfusion, and ventilation-perfusion matching in hyperinflated COPD patients, and that these imaging biomarkers would correlate with established physiological measures and cardiac MRI parameters reflecting lung deflation and improved cardiopulmonary interaction. By evaluating these functional proton MRI measures and comparing them with conventional cardiopulmonary outcomes and hyperpolarized ^129^Xe MRI, the present work aims to provide additional insight into how dual bronchodilation modifies regional lung physiology in hyperinflated COPD patients after SD and MD treatment.

## Methods

### Study design

This study represents a retrospective subanalysis of a prospective, single-center clinical trial approved by the local ethics committee. Written informed consent was obtained from all participants. The trial is registered at ClinicalTrials.gov (NCT04231214).

Patients aged ≥ 40 years with stable, moderate-to-severe COPD were recruited between June 2020 and April 2023. Inclusion criteria were: post-bronchodilator forced expiratory volume in one second (FEV₁) < 80% predicted after inhalation of 400 µg salbutamol, hyperinflation defined as residual volume (RV) > 135% pred, and FEV₁/FVC ratio < 0.7 (FVC: forced vital capacity). Eligible participants were current or former smokers with a smoking history of at least 10 pack-years.

**Fig. 1:**
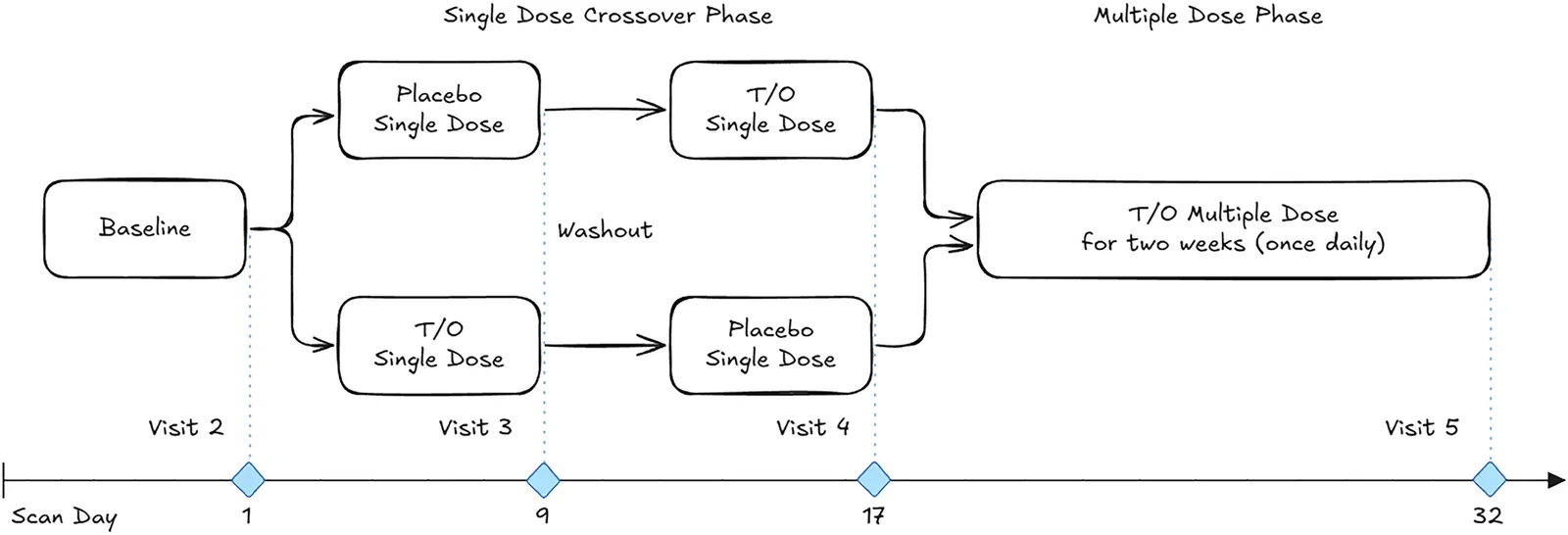
Study protocol. Each participant underwent pulmonary function tests (PFTs) and MR, including phase-resolved functional lung (PREFUL) imaging, at four study visits. After the baseline assessment (no medication) at visit 2, participants were randomized to receive either a single dose (SD) of placebo or tiotropium/olodaterol (T/O) prior to imaging (visit 3). Following a washout period of 7 ( ± 1) days, participants crossed over to the alternate single-dose treatment (visit 4). Subsequently, all participants entered a 2-week open-label phase of daily tiotropium/olodaterol multiple dose (MD) therapy, with final assessments performed at visit 5

Participants were randomized to one of two treatment sequences. Group A underwent: (visit 2) baseline scan without medication, (visit 3) scan after SD placebo (0.9% nebulized saline), (visit 4) scan after SD tiotropium bromide monohydrate/olodaterol hydrochloride (5 µg/5 µg; two inhalations, Spiolto® Respimat®, Boehringer Ingelheim, Ingelheim am Rhein, Germany), and (visit 5) a final scan after 2 weeks of once-daily home inhaler use, defined as MD in the following. A 7-day washout period was included between visits 2–4. Group B received the SD bronchodilator at visit 3 and placebo at visit 4, followed by the same 2-week open-label treatment as Group A, with the final measurements at visit 5. See Fig. 1 for an illustration of the study protocol.

### Assessments

In addition to assessments described and presented in the main study [46], including pulmonary function tests (PFTs), cardiac MRI, dynamic contrast enhanced (DCE) perfusion and hyperpolarized ^129^Xe MRI measurements, each visit included free-breathing PREFUL MRI. All MRI acquisitions were performed on a 1.5 T scanner (MAGNETOM Avanto, Siemens Healthineers).

PFTs were performed according to standardized protocols [47–49]. The parent trial included comprehensive testing comprising spirometry, body plethysmography, diffusion capacity, and impulse oscillometry [46]. For the present subanalysis, parameters reflecting airflow limitation and hyperinflation were selected, namely the FEV₁/FVC ratio and RV. The complete PFT dataset is provided in the Supplementary Material for reference.

Cardiac MRI was conducted using cine steady-state free precession sequences to assess ventricular volumes and function. Left and right ventricular (LV/RV) end-diastolic and end-systolic volumes (EDV, ESV), stroke volume (SV) and ejection fraction (EF) were quantified using semi-automated contouring software (cvi42, Circle Cardiovascular Imaging). Volumetric parameters were normalized to body surface area to yield indexed values (e.g., LV-EDVi, RV-ESVi).

PREFUL MRI was acquired in three coronal slices using a two-dimensional spoiled gradient-echo sequence with the central slice positioned at the tracheal bifurcation. The sequence parameters were as follows: field of view (FOV) 500 × 500 mm², slice thickness 15 mm, matrix 128  × 128 interpolated to 256 × 256, echo time (TE) 0.82 ms, repetition time (TR) 3 ms, flip angle (FA) 5°, parallel imaging acceleration factor 2, temporal resolution per frame 192 ms, slice distance factor 100%, and bandwidth 1500 Hz/pixel. The total acquisition time per slice was 60 s.

DCE MRI was performed following intravenous administration of a gadolinium-based contrast agent (0.04 mmol/kg bodyweight gadoteric acid bolus with an injection rate of 5 ml/s) using a dynamic (1.0–1.2 frames/second) spoiled gradient-echo sequence acquired during a single breath-hold with 30–36 reconstructed coronal sections (with a section thickness of 6 mm) covering the whole lung. Quantitative perfusion maps were generated from signal-time curves using model-based deconvolution [21], yielding voxel-wise estimates of pulmonary blood flow (PBF). For this subanalysis, the mean PBF of the lung parenchyma was included.

Hyperpolarized ^129^Xe MRI was performed using a dedicated xenon polarization system (9810, Polarean Inc.) and a 16-channel transmit-receive chest coil (Rapid Biomedical). Participants inhaled three 1 L gas bags containing different Xe/N₂ mixtures for calibration and imaging:

1. 35% xenon/65% nitrogen for calibration;
2. 50% xenon/50% nitrogen for chemical shift saturation recovery and diffusion-weighted imaging;
3. 60% xenon/40% nitrogen for dissolved-phase imaging, dynamic spectroscopy, and ventilation imaging.

Imaging was conducted during three consecutive single-breath-holds separated by several minutes of free breathing. Dose bags were inhaled from the functional residual capacity. Participants further inhaled room air after the second dose bag to reach full lung inflation.

For this subanalysis, the following parameters were derived: from ventilation imaging—the ventilation defect percentage Xe-VDP; from diffusion-weighted imaging—the apparent diffusion coefficient at 5000 μs (ADC₅₀₀₀_µs_); and from dissolved-phase imaging—signal ratios of the red blood cell (RBC), membrane (Mbr), and gas phases (GPs).

### PREFUL analysis

PREFUL MRI analysis was performed similarly as described previously [50]. The automated processing pipeline was implemented in MATLAB (R2021a, MathWorks) and executed on a dedicated analysis server. The software version was fixed at study initiation.

The following steps were performed after image acquisition:

#### Image registration

Non-rigid motion correction was carried out using the Advanced Normalization Tools framework [51]. Respiratory phase sorting was achieved by structural similarity analysis, with images grouped into 10 respiratory states. A group-oriented registration strategy [52] aligned all respiratory groups to a mid-position reference.

#### Preprocessing

Denoising was applied using image-guided filtering with the temporal mean as reference. Ventilation and perfusion signals were separated by frequency filtering (low-pass for ventilation, high-pass for perfusion). The first 20 images per slice were discarded to ensure steady state.

#### Segmentation

Lung boundaries were segmented on the temporal mean image using a pretrained convolutional neural network [53]. Central vessels were excluded by Otsu thresholding on perfusion-weighted maps, resulting in a parenchymal region of interest (ROI). Segmentations were visually reviewed by a radiologist (T.F.K., radiology resident with 7 years of pulmonary MRI experience at the time of analysis) and corrected if necessary.

#### Perfusion analysis

Cardiac phase sorting was based on a vessel-rich ROI determined iteratively from perfusion-weighted signal variation and sinusoidal fitting [54]. Phase-sorted images were interpolated onto an equidistant temporal grid with 15 cardiac phases. The parenchymal phase was determined from automated histogram analysis. QN was obtained by referencing parenchymal signal amplitudes to a vessel ROI in the tracheal slice.

#### Pulse-Wave velocity (PWV)

Using the dynamic information of the synthesized cardiac cycle, spatial distance and temporal delay between voxels located in the central and peripheral pulmonary vasculature were analyzed to determine pulmonary arterial PWV as previously described [55]. PWV analysis was exclusively conducted on the central slice. This parameter was evaluated post hoc, as it was not included in the fixed version of the PREFUL analysis at study initiation, but has recently been shown to correlate with hyperinflation and airflow obstruction in COPD [55].

#### Ventilation analysis

Respiratory phase assignment used unregistered signal fluctuations after percentile-based outlier exclusion and a cosine model. Phase-sorted images were interpolated onto an equidistant temporal grid with 60 respiratory phases. RVent was calculated as the relative signal change between end-expiration and end-inspiration [56]. The FVL-CM was derived by differentiating RVent over time to approximate airflow and cross-correlating voxel-wise flow-volume loops with a reference loop originating from a high-ventilation region.

#### Defect classification

Empirical threshold rules [57] were applied to define defective voxels: RVent < 40% of the 90th percentile, FVL-CM < 90%, and QN < 2%. Voxels below threshold were classified as ventilation or perfusion defects, and combined maps were used to derive ventilation-perfusion match (VQM) metrics. Quantitative parameters included the isolated ventilation defect percentage (VDP), derived separately from either RVent or FVL-CM, as well as a combined ventilation defect percentage, VDP(Combined), obtained by additively combining both VDPs. Additional parameters included the perfusion defect percentage (QDP) and the ventilation-perfusion quantitative match (VQM) for both defect and non-defect regions. For VQM, the VDP(Combined) and QDP measures were used.

#### Heterogeneity

In addition to mean values over the parenchymal ROI across all three slices, the coefficient of variation (CoV) was calculated to quantify intra-pulmonary heterogeneity of each parameter.

### Statistical analysis

When not stated otherwise, all statistical analyses were performed in MATLAB 2022b (MathWorks).

Baseline demographic and clinical characteristics were summarized as mean ± standard deviation (STD) for continuous variables and as absolute number (percentage) for categorical variables.

For the analysis of imaging and functional parameters, arithmetic means ± STD are reported alongside estimated contrasts between treatment conditions derived from linear mixed-effects models. The models included treatment, treatment sequence, and period as fixed effects and subject as a random effect, in line with the main trial publication. Estimated marginal means and two-sided 95% confidence intervals (CIs) were obtained using the emmeans framework in R (version 4.5.1, R Foundation for Statistical Computing).

Correlations between PREFUL MRI parameters, conventional cardiopulmonary measures, and hyperpolarized ^129^Xe MRI, as well as between treatment-induced changes, were assessed with Spearman’s rank correlation. For change analyses between T/O SD and placebo SD, the respective placebo SDs were included as confounding variables in a partial correlation [58]. CIs for correlation coefficients were estimated by bootstrap resampling (1000 iterations) and prioritized for inference due to limited sample size, which may lead to discrepancies with asymptotic *p*-values.

Analyses were performed using only available data, and no missing values were replaced or estimated.

Given the exploratory nature of the analysis, no statistical adjustment for multiple testing was applied. A two-sided α-level of 0.05 was considered statistically significant.

## Results

### Study population

A total of 32 patients with stable moderate-to-severe COPD were included in the analysis (17 males, 15 females; mean age ± STD: 61.5 ± 7.7 years). Baseline demographic and PREFUL characteristics are summarized in Table 1. All patients completed the crossover phase; after exclusion of two participants due to COVID-19 and a common cold, 30 patients completed the 2-week open-label extension.

**Table 1:** Baseline demographic and phase-resolved functional lung (PREFUL) MRI characteristics at visit 2 (pre-dose)

	Mean (STD)/*n* (%)
General	
Age (Years)	61.5 (7.7)
Male	17 (53%)
Female	15 (47%)
MI (kg/m²)	26.72 (4.29)
Current smokers	23 (72%)
Former smokers	9 (28%)
Pack years (Years)	55.4 (23.0)
Diastolic dysfunction (DD)	14 (44%)
Possible DD	11 (34%)
No DD	7 (22%)
Clinical	
GOLD 2	22 (69%)
GOLD 3	8 (25%)
GOLD 4	2 (6%)
Pulmonary function tests	
FEV1/FVC	0.41 (0.11)
RV (L)	4.13 (1.26)
PREFUL MRI	
RVent (%)	10.4 (5.2)
CoV(RVent) (%)	77.0 (21.1)
VDP(RVent) (%)	36.5 (14.4)
FVL-CM (%)	71.9 (14.5)
CoV(FVL-CM) (%)	60.3 (33.4)
VDP(FVL-CM) (%)	47.6 (18.6)
VDP(RVent & FVL-CM) (%)	55.0 (19.4)
QN (%)	2.7 (1.0)
CoV(QN) (%)	84.0 (18.2)
QDP (%)	45.4 (17.0)
VQM(Non-Defect) (%)	27.1 (17.4)
VQM(Defect) (%)	26.7 (16.1)
PWV (m/s)	2.48 (0.81)

Data are presented as mean (STD) or count (%)

*BMI* body mass index, *DD* diastolic dysfunction, *GOLD* global initiative for chronic obstructive lung disease, *FEV₁/FVC* Tiffeneau index, *RV* residual volume, *RVent* regional ventilation, *CoV* coefficient of variation, *FVL-CM* flow-volume correlation metric, *VDP* volume defect percentage, *QN* normalized perfusion, *QDP* perfusion defect percentage, *VQM* (non-defect) percentage of matched non-defect ventilation/perfusion regions, VQM (defect) percentage of matched defect ventilation/perfusion regions,* PWV* pulse wave velocity in the pulmonary arteries

### Linear mixed-model analysis

**Fig. 2:**
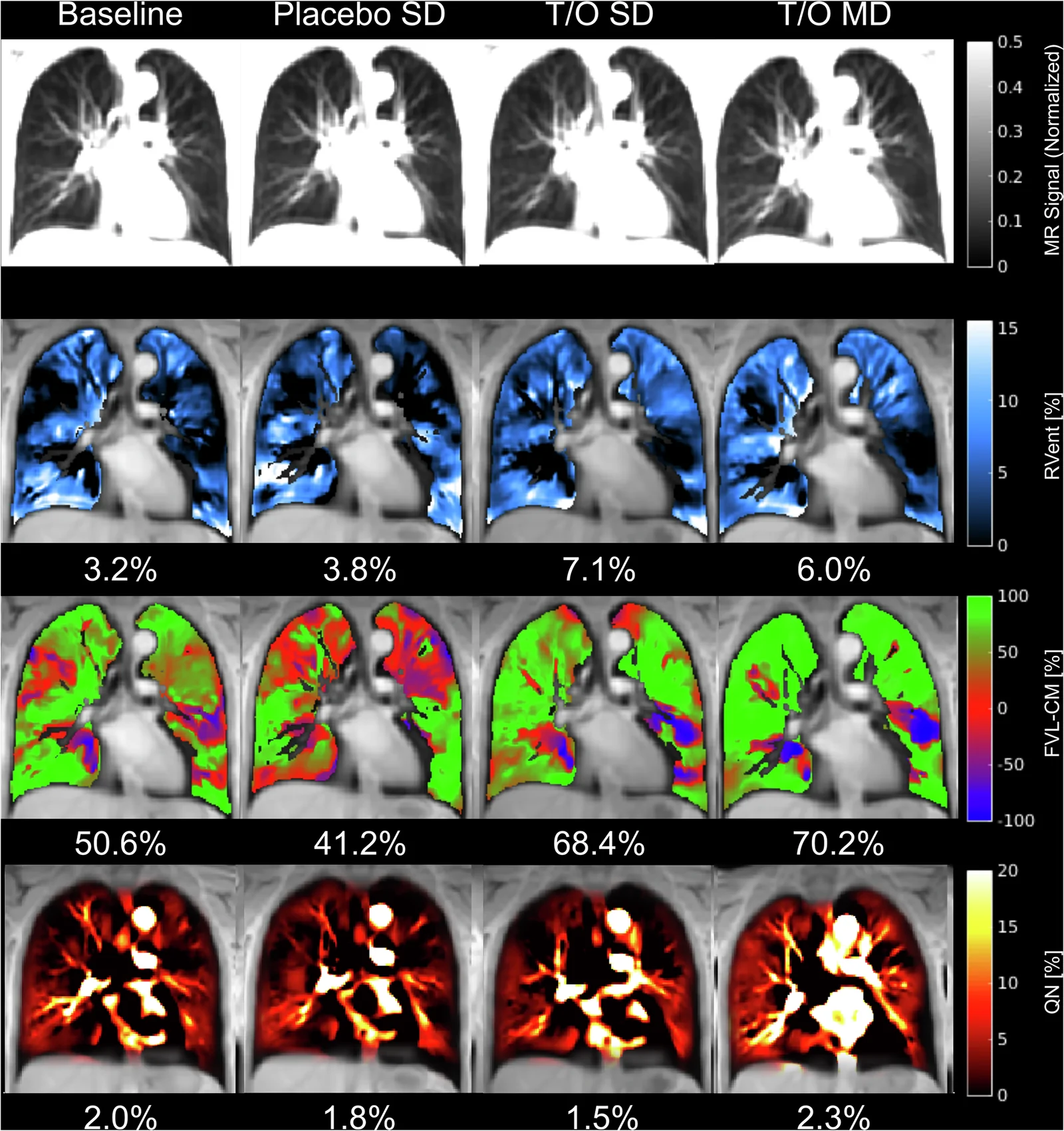
PREFUL MRI results in a 55-year-old female patient with chronic obstructive pulmonary disease (COPD). The first row shows temporally averaged, motion-corrected images from the spoiled gradient-echo acquisition, which served as the basis for deriving primary PREFUL parameters. The second to fourth rows depict regional ventilation (RVent), the flow-volume loop correlation metric (FVL-CM), and normalized perfusion (QN), respectively. Results are displayed across the four study visits: Baseline (visit 2), Placebo SD (visit 3), T/O SD (visit 4), and T/O MD (visit 5). Corresponding spirometric values (FEV₁% predicted; FEV₁/FVC) were: 24%, 0.30 (Baseline); 24%, 0.32 (Placebo SD); 37%, 0.30 (T/O SD); and 37%, 0.34 (T/O MD)

Representative PREFUL parameter maps from a single patient are shown in Fig. 2 with especially notable improvements in FVL-CM after single-dose bronchodilation and with further enhancement following multiple-dose therapy. Across the cohort, mean values and standard deviations of PREFUL parameters at each visit are provided in Table 2.

**Table 2:** Mean values ± standard deviations (Part A) and contrast estimates with 95% confidence intervals (Part B) for PREFUL-derived parameters

	Mean values ± standard deviations (Part A)				Contrast estimates with 95% confidence intervals (Part B)					
Outcome variable	Baseline	Placebo SD	T/O SD	T/O MD	T/O SD—Placebo SD	T/O MD—Placebo SD	T/O MD—T/O SD	Placebo SD—Baseline	T/O SD—Baseline	T/O MD—Baseline
RVent (%)	10.4 (5.2)	11.7 (6.2)	13.3 (5.7)	13.3 (6.0)	1.8 (0.7 to 3.0)**	1.5 (0.3 to 2.7)*	**−0.3** **(−1.5 to 1.0)**	1.0 (0.2 to 1.9)*	2.9 (1.5 to 4.3)***	2.6 (1.1 to 4.1)**
CoV(RVent) (%)	77.0 (21.1)	73.1 (19.7)	71.4 (16.4)	68.0 (17.1)	**−6.2** **(−24.3 to 12.0)**	**−2.5** **(−19.7 to 14.7)**	**1.9** **(−16.5 to 20.3)**	**−7.6** **(−19.4 to 4.2)**	**−13.8** **(−33.6 to 6.0)**	**−6.8** **(−20.0 to 6.4)**
VDP(RVent) (%)	36.5 (14.4)	34.9 (14.0)	32.9 (12.9)	31.3 (12.4)	**−2.0** **(−4.8 to 0.8)**	**−3.1** **(−7.0 to 0.7)**	**−1.3** **(−5.4 to 2.7)**	**−1.6** **(−4.0 to 0.9)**	−3.5 (−7.0 to −0.1)*	_4.8 (−8.6 to _0.9)*
FVL-CM (%)	71.9 (14.5)	71.8 (15.4)	76.3 (11.5)	79.7 (9.8)	4.1 (1.0 to 7.3)*	6.2 (2.7 to 9.7)**	**2.4** **(−0.0 to 4.8)**	**0.0** **(−2.7 to 2.7)**	4.2 (1.4 to 6.9)**	6.2 (3.1 to 9.3)***
CoV(FVL-CM) (%)	60.3 (33.4)	60.4 (36.2)	49.6 (23.7)	42.3 (21.2)	**−30.2** **(−61.6 to 1.1)**	−39.3 (−71.9 to −6.7)*	**−17.7** **(−37.0 to 1.6)**	**3.6** **(−12.5 to 19.8)**	**−26.6** **(−56.4 to 3.3)**	−40.8 (−71.3 to −10.3)**
VDP(FVL-CM) (%)	47.6 (18.6)	47.3 (18.1)	42.6 (16.4)	39.0 (14.0)	−5.4 (−9.7 to −1.2)*	−7.1 (−12.2 to −2.0)**	**−2.0** **(−5.8 to 1.7)**	**−0.3** **(−4.0 to 3.4)**	−5.7 (−9.4 to −2.0)**	−7.4 (−12.5 to −2.3)**
VDP(RVent&FVL-CM) (%)	55.0 (19.4)	54.5 (18.0)	49.8 (16.1)	46.8 (14.2)	−5.6 (−10.0 to −1.3)*	−7.0 (−11.9 to −2.1)**	**−1.8** **(−5.6 to 2.1)**	**−0.9** **(−4.9 to 3.1)**	−6.5 (−11.2 to −1.9)**	−7.6 (−12.5 to −2.6)**
QN (%)	2.7 (1.0)	2.5 (0.8)	2.9 (1.0)	2.8 (0.8)	0.4 (0.2 to 0.6)**	**0.2** **(_0.0 to 0.5)**	**−0.1** **(−0.4 to 0.1)**	**−0.2** **(−0.5 to 0.1)**	**0.2** **(−0.1 to 0.5)**	**0.0** **(−0.2 to 0.2)**
CoV(QN) (%)	84.0 (18.2)	85.3 (18.4)	87.7 (19.2)	84.1 (18.5)	**1.3** **(−2.3 to 4.9)**	**2.0** **(−2.3 to 6.2)**	**0.8** **(_1.1 to 2.7)**	**_1.1** **(−4.7 to 2.4)**	**0.2** **(−1.6 to 2.0)**	**1.0** **(−1.4 to 3.4)**
QDP (%)	45.4 (17.0)	48.6 (16.7)	43.6 (15.5)	43.8 (16.3)	−5.7 (−9.5 to −1.9)**	**−3.8** **(−8.9 to 1.3)**	**1.4** **(−3.4 to 6.2)**	**3.9** **(−1.2 to 9.1)**	**−1.8** **(−6.3 to 2.7)**	**0.5** **(−3.7 to 4.7)**
VQM(Defect) (%)	27.1 (17.4)	27.8 (17.7)	23.5 (14.3)	22.6 (14.2)	−4.2 (−6.8 to −1.6)**	**−3.9** **(−7.8 to 0.1)**	**−0.1** **(−3.6 to 3.4)**	**1.4** **(−1.9 to 4.8)**	**−2.8** **(−5.9 to 0.3)**	**−2.3** **(−6.1 to 1.5)**
VQM(Non-Defect) (%)	26.7 (16.1)	24.8 (13.6)	30.1 (14.3)	32.0 (15.0)	6.5 (3.4 to 9.6)***	6.4 (2.5 to 10.3)**	**0.5** **(−2.9 to 3.8)**	**−1.5** **(−4.6 to 1.5)**	4.9 (1.4 to 8.5)**	4.4 (1.2 to 7.7)**
PWV (m/s)	2.48 (0.81)	3.05 (1.14)	2.77 (1.09)	2.19 (0.72)	**−0.30** **(−0.63 to 0.03)**	−0.87 (−1.26 to −0.48)***	−0.57 (−0.90 to −0.25)**	0.57 (0.21 to 0.92)**	**0.27** **(−0.04 to 0.59)**	−0.28 (−0.65 to 0.08)
FEV_1_/FVC	0.41 (0.11)	0.41 (0.12)	0.42 (0.11)	0.45 (0.11)	**0.01** **(−0.01 to 0.03)**	0.03 (0.01 to 0.05)*	0.02 (0.01 to 0.03)**	**0.00** **(−0.01 to 0.01)**	**0.01** **(−0.00 to 0.03)**	0.03 (0.01 to 0.05)**
RV (L)	4.13 (1.26)	4.21 (1.31)	3.54 (1.21)	3.14 (0.77)	−0.67 (−0.82 to −0.51)***	−0.79 (−1.00 to −0.59)***	**−0.14** **(−0.33 to 0.05)**	**0.09** **(−0.05 to 0.24)**	−0.59 (−0.73 to −0.44)***	−0.77 (−0.97 to −0.56)***

Nearly all variables demonstrated significant differences between T/O SD and Placebo SD, characterized by improved functional metrics and reduced defect percentages following treatment. Please find the FEV₁/FVC and RV measures for reference in the lower part of the table. Non-significant contrast estimates are marked in bold font. Different levels of significance are reported with *p* < 0.05 (*), *p* < 0.01 (**) and *p* < 0.001 (***). Baseline values represent visit 2 pre-dose measurements, obtained before any study medication administration

#### Single-dose tiotropium/olodaterol versus placebo

Linear mixed-effects modeling showed significant differences between the T/O single dose and placebo for RVent, FVL-CM, and QN. FVL-CM was significantly increased after T/O treatment by 4.1 percentage points (pp; 95% CI: 1.0 to 7.3 pp, *p* = 0.01), while combined ventilation and perfusion defect percentages were significantly decreased (VDP(Combined): −5.6 pp, 95% CI: −10 to −1.3 pp, *p* = 0.01; QDP: −5.7 pp, 95% CI: −9.5 to −1.9 pp, *p* = 0.004). Similarly, VDP(FVL-CM) was also significantly decreased (−5.4 pp, 95% CI: −9.7 to −1.2 pp, *p* = 0.01), whereas VDP(RVent) alone was not significantly altered ( − 2.0 pp, 95% CI: −4.8 to 0.8 pp, *p* = 0.16). PWV showed a significant increase from baseline to the placebo visit but returned toward baseline values after single-dose T/O (*p* = 0.07).

#### Multiple-dose tiotropium/olodaterol

After multiple dosing, significant differences relative to placebo were observed for all PREFUL parameters except QN (*p* = 0.08). Compared with the single-dose condition, PWV showed a reduction of −0.57 m/s (95% CI: −0.90 to −0.25 m/s, *p *< 0.001). For the remaining PREFUL parameters, the estimated contrasts between multiple- and single-dose T/O were not statistically significant, although Cls for measures involving FVL-CM were shifted toward greater improvement.

**Fig. 3:**
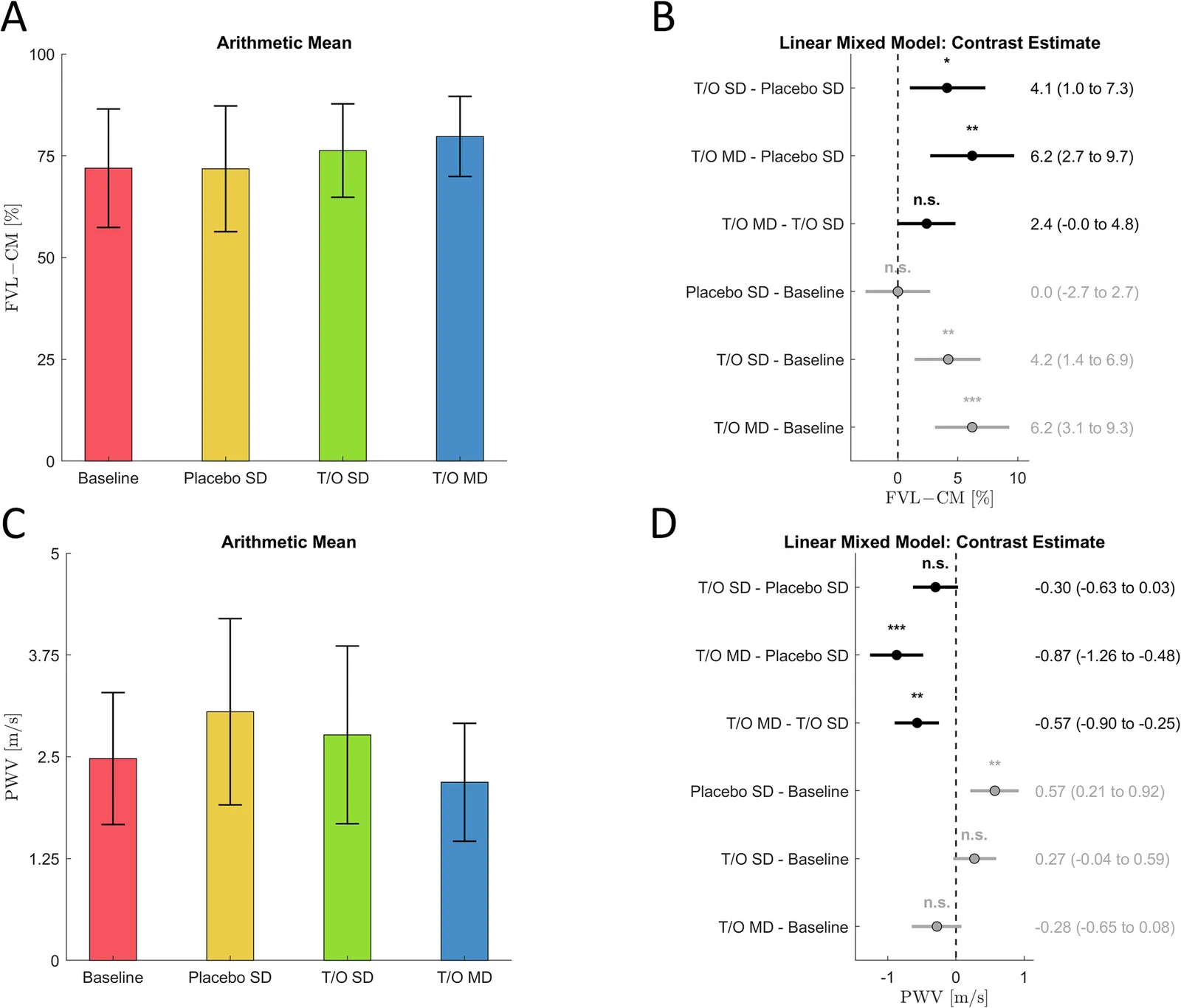
Arithmetic means and contrast estimates for FVL-CM and PWV. Top row (**A**, **B**): Arithmetic mean values ± STD of FVL-CM across all treatment conditions (**A**) and corresponding contrast estimates with 95% confidence intervals from the linear mixed-effects model (**B**). Bottom row (**C**, **D**): Arithmetic mean values ± STD of pulmonary pulse-wave velocity (PWV) across all treatment conditions (**C**) and corresponding contrast estimates (**D**). Different levels of significance are reported with *p* < 0.05 (*), *p* < 0.01 (**) and *p* < 0.001 (***)

Results for selected PREFUL endpoints are illustrated in Fig. 3, with estimated contrasts and CIs for all endpoints summarized in Table 2.

### Correlation analyses

At baseline (Table 3), all PREFUL parameters were correlated with FEV1/FVC and RV. For instance, VDP(Combined) with FEV1/FVC: *ρ* = −0.71, 95% CI: −0.88 to −0.40; and VQM(Defect) with RV: *ρ* = 0.82, 95% CI: 0.63 to 0.92. QN and derived parameters correlated with PBF, e.g., QN with PBF: *ρ* = 0.39, 95% CI: 0.02 to 0.66. QN was also correlated with LV-EDVi: *ρ* = 0.40, 95% CI: 0.04 to 0.68. Except for RVent and PWV, all parameters were correlated with Xe-VDP and Mbr/GP ratio, e.g., VQM(Non-Defect) with Mbr/GP: *ρ* = 0.68, 95% CI: 0.38 to 0.83. ADC₅₀₀₀_µs_ showed a strong correlation with QDP: *ρ* = 0.60, 95% CI: 0.29 to 0.77.

**Table 3:** Spearman correlations between PREFUL MRI parameters and spirometry, body plethysmography, cardiac MRI, and hyperpolarized ^129^Xe MRI at baseline

Variable	FEV_1_/FVC	RV (L)	PBF (mL/min/100 mL)	LV-EDVi (mL/m²)	Xe-VDP (%)	ADC_5000μs_ (mm²/s)	Mbr/GP (%)
RVent (%)	0.41 (0.04 to 0.68)*	−0.38 (−0.63 to −0.05)*	**−0.02** **(−0.38 to 0.35)**	**−0.00** **(−0.39 to 0.36)**	**−0.18** **(−0.49 to 0.18)**	**0.05** **(−0.29 to 0.40)**	**0.30** **(−0.04 to 0.58)**
CoV(RVent) (%)	−0.68 (−0.85 to −0.39)***	0.70 (0.49 to 0.83)***	**−0.09** **(−0.40 to 0.26)**	**−0.09** **(−0.41 to 0.28)**	0.57 (0.22 to 0.76)**	**0.20** **(−0.17 to 0.51)**	−0.55 (−0.79 to −0.15)**
VDP(RVent) (%)	−0.71 (−0.84 to −0.48)***	0.75 (0.54 to 0.86)***	**−0.20** **(−0.52 to 0.18)**	**−0.11** **(−0.43 to 0.27)**	0.62 (0.31 to 0.80)***	**0.32** **−**	−0.66 (−0.87 to −0.34)***
FVL-CM (%)	0.74 (0.46 to 0.88)***	−0.76 (−0.89 to −0.55)***	**0.15** **(−0.19 to 0.46)**	**0.10** **(−0.27 to 0.40)**	-0.59 (−0.79 to −0.28)***	**−0.30** **(−0.59 to 0.06)**	0.65 (0.30 to 0.85)***
CoV(FVL-CM) (%)	−0.70 (−0.85 to −0.43)***	0.73 (0.45 to 0.87)***	**−0.18** **(−0.49 to 0.18)**	**−0.07** **(−0.38 to 0.31)**	0.56 (0.22 to 0.77)**	**0.26** **(−0.09 to 0.54)**	−0.69 (−0.87 to −0.31)***
VDP(FVL-CM) (%)	−0.74 (−0.89 to −0.45)***	0.78 (0.58 to 0.90)***	**−0.21** **(−0.53 to 0.15)**	**−0.19** **(−0.48 to 0.18)**	0.65 (0.34 to 0.84)***	0.38 (0.06 to 0.65)*	−0.62 (−0.82 to −0.31)***
VDP(RVent&FVL-CM) (%)	−0.71 (−0.88 to −0.40)***	0.78 (0.59 to 0.89)***	**−0.18** **(−0.51 to 0.16)**	**−0.17** **(−0.47 to 0.21)**	0.63 (0.30 to 0.82)***	**0.33** **(−0.01 to 0.62)**	−0.58 (−0.81 to −0.24)***
QN (%)	0.60 (0.30 to 0.78)***	−0.59 (−0.79 to −0.28)***	0.39 (0.02 to 0.66)*	0.40 (0.04 to 0.68)*	**−0.39** (−0.70 to 0.01)*	−0.58 (−0.74 to −0.28)***	0.61 (0.31 to 0.81)***
CoV(QN) (%)	−0.59 (−0.80 to −0.30)***	0.71 (0.41 to 0.87)***	−0.38 (−0.65 to −0.03)*	**−0.26** **(−0.52 to 0.08)**	0.50 (0.18 to 0.74)**	0.55 (0.23 to 0.77)**	−0.60 (−0.78 to −0.30)***
QDP (%)	−0.60 (−0.80 to −0.32)***	0.64 (0.32 to 0.85)***	−0.39 (−0.66 to −0.03)*	**−0.34** **(−0.64 to 0.06)**	0.44 (0.04 to 0.73)*	0.60 (0.29 to 0.77)***	−0.63 (−0.82 to −0.33)***
VQM(Defect) (%)	−0.74 (−0.89 to −0.50)***	0.82 (0.63 to 0.92)***	**−0.33** **(−0.60 to 0.00)**	**−0.28** **(−0.57 to 0.07)**	0.62 (0.31 to 0.80)***	0.49 (0.18 to 0.68)**	−0.67 (−0.85 to −0.40)***
VQM(Non-Defect) (%)	0.71 (0.44 to 0.86)***	−0.80 (−0.90 to −0.58)***	**0.33** **(−0.04 to 0.60)**	**0.25** **(−0.14 to 0.55)**	−0.64 (−0.81 to −0.33)***	−0.48 (−0.71 to −0.16)**	0.68 (0.38 to 0.83)***
PWV (m/s)	−0.38 (−0.66 to −0.01)*	0.45 (0.10 to 0.72)*	**−0.08** **(−0.48 to 0.34)**	**−0.26** **(−0.53 to 0.07)**	0.55 (0.24 to 0.78)**	**0.23** **(−0.19 to 0.59)**	**−0.24** **(−0.55 to 0.13)**

Correlation coefficients are reported with 95% confidence intervals in brackets. Spirometry and body plethysmography parameters included FEV₁/FVC (Tiffeneau index) and RV (residual volume). From perfusion MRI, PBF (pulmonary blood flow) was included. As cardiac MRI measures LV-EDVi (left ventricular end-diastolic volume index), it was included. Hyperpolarized ^129^Xe MRI parameters included VDP (ventilation defect percentage), ADC₅₀₀₀_µs_ (apparent diffusion coefficient at 5000 µs) and the dissolved-phase ratio Mbr/GP (membrane/gas phase) and RBC/GP (red blood cell/gas phase). For clarity of presentation, only variables with at least one significant correlation are included in the table. Levels of significance are reported with *p* < 0.05 (*), *p* < 0.01 (**) and *p* < 0.001 (***). Non-significant or correlations with zero-crossing are shown in bold font

SD treatment-induced changes versus placebo (Table 4) consistently involved ΔFVL-CM and derived measurements as key correlates. For instance, ΔVDP(FVL-CM) was correlated with reductions in ΔRV (*ρ* = 0.41, 95% CI: 0.02 to 0.64) and ΔFVL-CM with improvements in ΔMbr/GP (*ρ* = 0.48, 95% CI: 0.19 to 0.69) and ΔRBC/GP ratio (*ρ* = 0.55, 95% CI: 0.24 to 0.77). As for baseline, ΔQDP was positively correlated with the ΔADC₅₀₀₀_µs_: *ρ* = 0.50, 95% CI: 0.17 to 0.77.

**Table 4:** Partial Spearman correlations were computed between absolute changes in PREFUL MRI parameters and corresponding changes in spirometry, body plethysmography, cardiac MRI, and hyperpolarized ^129^Xe MRI between T/O SD and placebo SD treatments

Variable	ΔRV (L)	ΔXe-VDP (%)	ΔADC_5000μs_ (mm²/s)	ΔMbr/GP (%)	ΔRBC/GP (%)
ΔRVent (%)	**−0.24** **(−0.55 to 0.15)**	**−0.15** **(−0.51 to 0.30)**	**0.09** **(−0.31 to 0.48)**	**0.23** **(−0.19 to 0.62)**	**−0.02** **(−0.37 to 0.41)**
ΔCoV(RVent) (%)	**0.02** **(−0.37 to 0.39)**	**0.27** **(−0.15 to 0.60)**	**−0.27** **(−0.63 to 0.13)**	**−0.13** **(−0.49 to 0.27)**	**-0.17** **(−0.53 to 0.22)**
ΔVDP(RVent) (%)	**0.18** **(−0.23 to 0.57)**	**0.30** **(−0.11 to 0.65)**	**−0.27** **(−0.64 to 0.10)**	**−0.26** **(−0.61 to 0.06)**	**−0.33** **(−0.64 to 0.05)**
ΔFVL-CM (%)	**−0.38** (−0.69 to 0.03)*	**−0.44** (−0.77 to 0.04)*	**0.13** **(−0.30 to 0.51)**	0.48 (0.19 to 0.69)**	0.55 (0.24 to 0.77)**
ΔCoV(FVL-CM) (%)	**0.24** **(−0.17 to 0.60)**	0.47 (0.00 to 0.78)*	**−0.13** **(−0.45 to 0.25)**	−0.43 (−0.67 to −0.14)*	−0.52 (−0.76 to −0.22)**
ΔVDP(FVL-CM) (%)	0.41 (0.02 to 0.64)*	**0.23** **(−0.17 to 0.55)**	**−0.22** **(−0.53 to 0.20)**	−0.48 (−0.70 to −0.15)**	−0.46 (−0.71 to −0.06)*
ΔVDP(RVent&FVL-CM) (%)	**0.34** **(0.00 to 0.61)**	**0.34** **(−0.06 to 0.68)**	**−0.31** **(−0.58 to 0.06)**	−0.52 (−0.76 to −0.17)**	−0.43 (−0.72 to −0.10)*
ΔQN (%)	**0.15** **(−0.24 to 0.47)**	**−0.01** **(−0.40 to 0.42)**	−0.54 (−0.76 to −0.20)**	**−0.11** **(−0.44 to 0.20)**	**−0.16** **(−0.54 to 0.18)**
ΔCoV(QN) (%)	**0.24** **(−0.15 to 0.61)**	**−0.12** **(−0.51 to 0.24)**	**0.18** **(−0.22 to 0.55)**	**0.05** **(−0.38 to 0.45)**	**0.03** **(−0.47 to 0.45)**
ΔQDP (%)	**−0.09** **(−0.45 to 0.29)**	**0.04** **(−0.40 to 0.47)**	0.50 (0.17 to 0.77)**	**0.16** **(−0.21 to 0.56)**	**0.17** **(−0.23 to 0.54)**
ΔVQM(Defect) (%)	**0.17** **(−0.25 to 0.54)**	**0.35** **(−0.12 to 0.71)**	**0.32** **(−0.08 to 0.62)**	**−0.33** **(−0.64 to 0.11)**	**−0.30** **(−0.63 to 0.08)**
ΔVQM(Non-Defect) (%)	**−0.25** **(−0.53 to 0.10)**	**−0.31** **(−0.60 to 0.05)**	**−0.11** **(−0.48 to 0.29)**	**0.39** (−0.05 to 0.71)*	**0.33** **(−0.04 to 0.61)**
ΔPWV (m/s)	**−0.19** **(−0.56 to 0.27)**	**−0.32** **(−0.72 to 0.16)**	**−0.32** **(−0.65 to 0.11)**	0.54 (0.22 to 0.75)**	0.40 (0.02 to 0.68)*

Correlations were adjusted for the placebo SD measurement as a confounding variable. All tested Parameters and their definitions are provided in Table 3. For clarity of presentation, only selected variables are included in the table. Levels of significance are reported with *p* < 0.05 (*) and *p* < 0.01 (**).

**Fig. 4:**
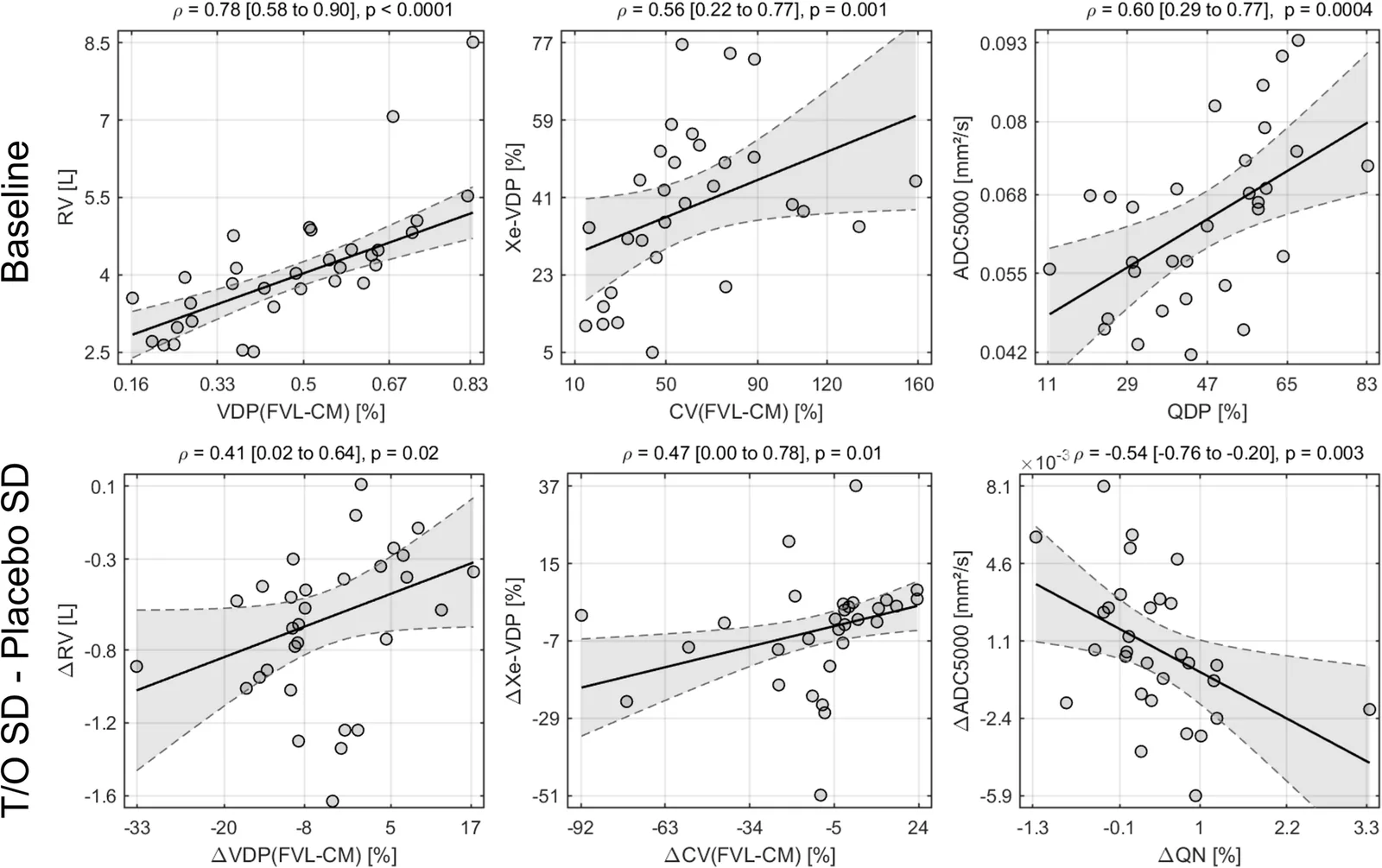
Example correlation plots. Top row: Baseline correlations. Bottom row: Contrast Δ(SD) = T/O single dose −  Placebo single dose. Shown are correlations of VDP(FVL-CM) with RV, CV(FVL-CM) with hyperpolarized ^129^Xe ventilation defect percentage (Xe-VDP) and QDP with the apparent diffusion coefficient at 5000 μs (ADC_5000μs_). Each dot represents one participant. Solid lines indicate robust linear fits with shaded 95% CIs; Spearman’s ρ with bootstrap CIs and *p*-values are given in the panels

No significant correlations were found with SD treatment-induced changes in DCE or cardiac MRI parameters. Example correlation plots are shown in Fig. 4.

## Discussion

This subanalysis extends the findings of the randomized, placebo-controlled crossover trial of T/O in hyperinflated COPD patients [46] by incorporating PREFUL MRI. In concordance with the main trial, PREFUL parameters demonstrated immediate improvements in V/Q alongside further pulmonary vascular improvements with continued medication. Thus, the current study provides novel evidence that PREFUL-derived parameters capture both acute and sustained treatment effects of dual bronchodilation.

The linear mixed-model analysis showed consistent improvements in RVent, FVL-CM, and QN following single-dose T/O. These changes were paralleled by reductions in defect percentages (VDP, QDP) and improvements in ventilation-perfusion matching (VQM). Although FVL-CM and its derived VDP metrics showed numerically greater improvements after two weeks of sustained treatment compared with placebo, these differences did not reach statistical significance when directly compared with the single-dose results, indicating the predominance of immediate improvements in lung function after SD.

In contrast, a rise in pulmonary PWV was observed from baseline to the single-dose placebo visit, whereas PWV at both T/O single-dose and multiple-dose did not differ from baseline. A similar “worsening under placebo” pattern was noted for LV-EDVi in the main trial results. Given that regional pulmonary ventilation parameters did not deteriorate after saline inhalation, a pure nocebo effect seems unlikely. The most plausible explanation is incomplete medication wash-out prior to the baseline visit, which may have led to apparent worsening in the eight-day time interval between baseline and placebo. However, the absence of this effect in other parameters remains unexplained.

Notably, sustained T/O dosing reduced PWV relative to single-dose T/O, consistent with a time-dependent vascular adaptation once lung deflation is maintained. Together, these findings indicate that PREFUL-derived PWV is sufficiently sensitive to detect reduced pulmonary artery pulse wave velocities after T/O treatment, likely reflecting reduced pulmonary vascular resistance due to improved pulmonary capillary blood flow in response to improved oxygenation due to improved ventilation [59].

At baseline, PREFUL V/Q parameters correlated strongly with established markers of airflow limitation and hyperinflation, confirming their validity as functional surrogates. More importantly, when comparing treatment-induced changes between placebo and T/O single dose, the correlation of FVL-CM parameters with RV persisted, supporting the sensitivity of dynamic regional tidal-volume flow-volume loop parameters of PREFUL to bronchodilation effects of a single T/O dose.

Baseline associations with cardiac MRI were less frequent but consistently involved PREFUL-derived perfusion measurements, consistent with the notion that impaired pulmonary perfusion limits ventricular filling and ejection [60].

In line with previous validation studies [36, 54, 57, 61], baseline PREFUL-derived perfusion (QN) correlated with DCE perfusion (PBF). Although both techniques showed similar treatment-response patterns, with significant improvement after single-dose T/O compared with placebo, subject-level changes in QN did not correlate with PBF, suggesting that while the group-level trends are consistent, individual variability and methodological differences limit direct comparability.

DCE perfusion provides absolute, volumetric first-pass contrast kinetics at relatively high spatial resolution, whereas PREFUL perfusion reflects cyclic signal oscillations normalized to a reference region and was restricted to three coronal slices in this study. These differences in spatial resolution, lung coverage, and normalization may explain the lack of subject-level correlation, and further refinement of PREFUL perfusion quantification [62] toward volumetric coverage may improve correspondence with DCE.

As anticipated by previous work [37, 45], PREFUL-MRI parameters correlated with ^129^Xe VDP. Notably, PREFUL parameters also correlated with diffusion metrics and pulmonary compartment ratios, which probe microstructural and gas-transfer properties not directly accessible with proton MRI. These correlations likely arise from shared pathophysiological substrates: regions of microstructural damage or impaired gas transfer also manifest as V/Q abnormalities detectable by PREFUL. Thus, although PREFUL does not directly assess alveolar microstructure, its regional functional parameters may indirectly track microstructural disease burden. Similar indirect connections between xenon transfer metrics and PFT measures have been reported [63], supporting this interpretation.

In addition, the observed correlations between SD treatment-induced changes in PREFUL parameters and Xe-VDP, diffusion and compartment measures highlight the potential of contrast-agent-free proton MRI to approximate ventilation, microstructural and gas-exchange-related information, thereby extending its translational value for interventional COPD studies.

Our findings align with earlier bronchodilator studies involving PREFUL. In severe asthma, Friedlander et al recently showed that PREFUL-derived VDP(RVent) was significantly elevated compared with healthy controls and decreased after bronchodilator therapy, correlating with spirometry, oscillometry, and ^129^Xe MRI [64]. Their study highlighted PREFUL VDP as a responsive, physiologically relevant outcome in asthma. In COPD, Voskrebenzev et al retrospectively analyzed the CLAIM trial and identified FVL-CM as the PREFUL parameter most strongly responsive to 14-day indacaterol-glycopyrronium therapy. Similar to the present study, they reported correlations between treatment-induced changes in LV-EDV and VQM, supporting the link between V/Q improvements and cardiac adaptation.

Taken together, these results extend the understanding of dual bronchodilation. PREFUL not only detected acute improvements in V/Q but also tracked, especially, longer-term changes in vascular adaptation. The correlations at baseline and for SD treatment changes of PREFUL-derived parameters with both conventional lung function tests and ^129^Xe MRI highlight its translational value as a non-invasive biomarker for COPD studies.

Limitations include the single-center design, relatively small sample size, and the exploratory nature of multiple correlation analyses without correction for multiplicity. A small proportion of the reported correlations showed CIs crossing zero or unexpected directionality, suggesting the possibility of random findings. Nevertheless, most correlations were physiologically plausible and consistent with previous literature. Regarding acquisition, PREFUL was limited to three coronal slices and may underestimate regional heterogeneity. Future studies should validate whole-lung acquisitions and evaluate PREFUL in larger interventional cohorts.

In conclusion, free-breathing PREFUL MRI detected improvements in regional ventilation, perfusion, and V/Q matching following a single dose of dual bronchodilation in COPD patients, and captured normalization of pulmonary arterial pulse wave velocity, likely reflecting reduction of pulmonary vascular resistance after 2 weeks of treatment.

## Supplementary information

Supplementary information

## Abbreviations

¹²⁹Xe: Xenon-129 (hyperpolarized gas)
³He: Helium-3 (hyperpolarized gas)
ADC₅₀₀₀µs: Apparent diffusion coefficient at 5000 μs
CI: Confidence interval
COPD: Chronic obstructive pulmonary disease
CoV: Coefficient of variation
DCE: Dynamic contrast-enhanced (MRI)
EDV: End-diastolic volume
EF: Ejection fraction
ESV: End-systolic volume
FA: Flip angle
FEV₁: Forced expiratory volume in one second
FVC: Forced vital capacity
FVL-CM: Flow-volume loop correlation metric
GP: Gas phase
LABA: Long-acting β₂-agonists
LAMA: Long-acting muscarinic antagonists
LV/RV: Left ventricle/right ventricle
LV-EDVi: Left ventricular end-diastolic volume index
Mbr: Membrane
MD: Multiple dose
NCT: National Clinical Trial (registry number)
PBF: Pulmonary blood flow
PFTs: Pulmonary function tests
PREFUL: Phase-resolved functional lung (MRI)
PWV: Pulse-wave velocity
QDP: Perfusion defect percentage
QN: Normalized perfusion
RBC: Red blood cell
ROI: Region of interest
RVent: Regional ventilation
SD: Single dose
STD: Standard deviation
SV: Stroke volume
SVi: Stroke volume index
T/O: Tiotropium/olodaterol
TE: Echo time
TR: Repetition time
V/Q: Ventilation-perfusion
VDP: Ventilation defect percentage
VDP(Combined): Combined ventilation defect percentage
VQM: Ventilation-perfusion match percentage
Xe-VDP: Xenon ventilation defect percentage
Δ: Change/delta
