# Supplementary material for: Phase-resolved functional lung MRI detects single-dose and sustained bronchodilator responses in COPD in a randomized crossover trial
Source: Eur Radiol. 2026 May 20;36(10):7731–46. doi: 10.1007/s00330-026-12604-6 (PMC13574893; doi:10.1007/s00330-026-12604-6)
Supplement: Supplementary file 1 [file 330_2026_12604_MOESM1_ESM.pdf]

Phase-resolved functional lung MRI detects single-dose and sustained bronchodilator responses in COPD in a randomized crossover trial

ELECTRONIC SUPPLEMENTARY MATERIAL

Supplementary Table 1: Mean values ± standard deviations (Part A) and contrast estimates with 95% confidence intervals (Part B) for following parameters: Spirometry - FEV1 (forced expiratory volume in 1 second), FVC (forced vital capacity), FEV_1/FVC ratio (Tiffeneau index), PEF (peak expiratory flow), $MFEF_{25-75}$ (mean forced expiratory flow between 25% and 75% of FVC). Body plethysmography - RV (residual volume), TLC (total lung capacity), RV/TLC (ratio of residual volume to total lung capacity), IC (inspiratory capacity), SVC ex (slow vital capacity expiration), sReff (specific effective airway resistance). Diffusing capacity - DLCOc SB (single-breath diffusing capacity for carbon monoxide corrected for hemoglobin), KCO (transfer coefficient of the lung for carbon monoxide), VA (alveolar volume). Impulse oscillometry - R_{5-20} (resistance difference between 5 Hz and 20 Hz), AX (area of reactance). Non-significant contrast estimates are marked in gray. Different levels of significance are reported with $p < 0.05$ (*), $p < 0.01$ (**) and $p < 0.001$ (***). Baseline values represent visit 2 pre-dose measurements, obtained before any study medication administration.

	**Mean Values ± standard deviations (Part A)**				**Contrast Estimates with 95% confidence intervals (Part B)**					
**Outcome Variable**	**Baseline**	**Placebo SD**	**T/O SD**	**T/O MD**	**T/O SD – Placebo SD**	**T/O MD – Placebo SD**	**T/O MD – T/O SD**	**Placebo SD – Baseline**	**T/O SD – Baseline**	**T/O MD – Baseline**
**FEV1 [L]**	1.36 (0.50)	1.37 (0.56)	1.63 (0.58)	1.75 (0.61)	0.27 (0.20 to 0.34)***	0.36 (0.28 to 0.44)***	0.08 (0.02 to 0.14)**	-0.01 (-0.06 to 0.05)	0.27 (0.20 to 0.33)***	0.36 (0.28 to 0.44)***
**FVC [L]**	3.34 (0.89)	3.31 (0.95)	3.85 (0.90)	3.90 (0.98)	0.56 (0.44 to 0.68)***	0.60 (0.48 to 0.73)***	0.04 (-0.05 to 0.14)	-0.05 (-0.15 to 0.04)	0.51 (0.40 to 0.61)***	0.57 (0.47 to 0.67)***
**FEV1/FVC**	0.41 (0.11)	0.41 (0.12)	0.42 (0.11)	0.45 (0.11)	0.01 (-0.01 to 0.03)	0.03 (0.01 to 0.05)*	0.02 (0.01 to 0.03)**	0.00 (-0.01 to 0.01)	0.01 (-0.00 to 0.03)	0.03 (0.01 to 0.05)**
**PEF [L/s]**	3.98 (1.41)	4.03 (1.63)	4.76 (1.55)	4.98 (1.70)	0.80 (0.64 to 0.96)***	1.00 (0.81 to 1.20)***	0.19 (0.00 to 0.37)*	-0.00 (-0.21 to 0.21)	0.79 (0.56 to 1.02)***	0.98 (0.75 to 1.21)***
**$MFEF_{25-75}$ [L/s]**	0.37 (0.22)	0.38 (0.18)	0.46 (0.20)	0.49 (0.24)	0.09 (0.05 to 0.13)***	0.10 (0.04 to 0.17)**	0.01 (-0.03 to 0.06)	0.00 (-0.05 to 0.06)	0.09 (0.03 to 0.15)**	0.11 (0.03 to 0.18)**
**RV [L]**	4.13 (1.26)	4.21 (1.31)	3.54 (1.21)	3.14 (0.77)	-0.67 (-0.82 to -0.51)***	-0.79 (-1.00 to -0.59)***	-0.14 (-0.33 to 0.05)	0.09 (-0.05 to 0.24)	-0.59 (-0.73 to -0.44)***	-0.77 (-0.97 to -0.56)***
**RV [%pred]**	186.31 (44.32)	189.25 (45.73)	159.29 (41.16)	145.62 (29.97)	-30.77 (-37.67 to -23.86)***	-36.92 (-45.93 to -27.91)***	-6.26 (-14.25 to 1.73)	4.39 (-2.00 to 10.77)	-27.02 (-33.66 to -20.38)***	-35.20 (-43.96 to -26.43)***
**TLC [L]**	7.37 (1.51)	7.42 (1.44)	7.27 (1.39)	6.97 (1.26)	-0.13 (-0.24 to -0.03)*	-0.19 (-0.34 to -0.05)**	-0.08 (-0.22 to 0.06)	0.02 (-0.11 to 0.15)	-0.11 (-0.24 to 0.02)	-0.17 (-0.35 to -0.00)*
**RV / TLC**	0.56 (0.09)	0.56 (0.10)	0.48 (0.09)	0.45 (0.08)	-0.08 (-0.10 to -0.07)***	-0.10 (-0.12 to -0.08)***	-0.01 (-0.03 to 0.01)	0.01 (-0.00 to 0.03)	-0.07 (-0.09 to -0.06)***	-0.09 (-0.11 to -0.07)***
**IC [L]**	2.23 (0.67)	2.21 (0.71)	2.55 (0.73)	2.62 (0.81)	0.35 (0.25 to 0.46)***	0.38 (0.26 to 0.49)***	0.02 (-0.05 to 0.09)	-0.04 (-0.11 to 0.03)	0.32 (0.23 to 0.42)***	0.37 (0.26 to 0.48)***
**SVC ex [L]**	3.22 (0.81)	3.20 (0.90)	3.71 (0.86)	3.80 (0.98)	0.53 (0.42 to 0.64)***	0.57 (0.43 to 0.71)***	0.04 (-0.07 to 0.15)	-0.05 (-0.17 to 0.06)	0.49 (0.37 to 0.61)***	0.59 (0.46 to 0.71)***

**sReff [kPa s]**	3.74 (2.03)	3.78 (2.04)	2.08 (1.33)	1.81 (1.17)	-1.72 (-2.19 to -1.24)***	-1.69 (-2.11 to -1.27)***	-0.04 (-0.25 to 0.16)	0.11 (-0.11 to 0.33)	-1.66 (-2.12 to -1.20)***	-1.68 (-2.13 to -1.23)***
**DLCOc SB [mmol/min/kPa]**	5.44 (1.65)	5.58 (1.77)	5.24 (1.73)	5.47 (1.66)	-0.09 (-0.27 to 0.08)	0.08 (-0.13 to 0.30)	0.20 (0.05 to 0.35)*	0.06 (-0.09 to 0.20)	-0.06 (-0.20 to 0.07)	0.19 (0.03 to 0.34)*
**KCO [mmol/min/kPa/L]**	1.00 (0.28)	1.00 (0.27)	0.96 (0.27)	0.97 (0.27)	-0.03 (-0.05 to -0.01)*	-0.04 (-0.06 to -0.01)*	0.00 (-0.02 to 0.03)	0.00 (-0.02 to 0.02)	-0.03 (-0.05 to -0.01)**	-0.03 (-0.05 to -0.01)*
**VA [L]**	5.52 (0.99)	5.60 (1.01)	5.51 (1.02)	5.72 (1.05)	0.10 (-0.02 to 0.22)	0.29 (0.15 to 0.44)***	0.19 (0.08 to 0.29)***	0.02 (-0.10 to 0.13)	0.07 (-0.07 to 0.22)	0.32 (0.21 to 0.43)***
**R_{5-20} [kPa/L/s]**	0.45 (0.09)	0.46 (0.09)	0.39 (0.09)	0.41 (0.10)	-0.07 (-0.09 to -0.05)***	-0.06 (-0.08 to -0.04)***	0.01 (-0.01 to 0.03)	0.02 (-0.00 to 0.04)	-0.06 (-0.08 to -0.04)***	-0.05 (-0.07 to -0.02)***
**AX (Hz kPa/L/s)**	3.35 (1.48)	3.38 (1.60)	1.78 (1.10)	1.75 (1.22)	-1.64 (-2.00 to -1.28)***	-1.63 (-1.99 to -1.28)***	-0.01 (-0.26 to 0.23)	0.10 (-0.16 to 0.36)	-1.57 (-1.90 to -1.25)***	-1.63 (-2.04 to -1.23)***
